# Huber’s Non-Linearity for GNSS Interference Mitigation †

**DOI:** 10.3390/s18072217

**Published:** 2018-07-10

**Authors:** Daniele Borio, Haoqing Li, Pau Closas

**Affiliations:** 1 European Commission, Joint Research Centre (JRC) Directorate for Space, Security and Migration; Via Enrico Fermi 2749, 21027 Ispra (VA), Italy; 2 Northeastern University, Electrical and Computer Engineering Department, 360 Huntington Ave, Boston, MA 02115, USA; li.haoq@husky.neu.edu (H.L.); closas@northeastern.edu (P.C.)

**Keywords:** GNSS, Huber’s non-linearity, interference, jamming, robustness, M-estimator

## Abstract

Satellite-based navigation is prevalent in both commercial applications and critical infrastructures, providing precise position and time referencing. As a consequence, interference to such systems can have repercussions on a plethora of fields. Additionally, Privacy Preserving Devices (PPD)—jamming devices—are relatively inexpensive and easy to obtain, potentially denying the service in a wide geographical area. Current jamming mitigation technology is based on interference cancellation approaches, requiring the detection and estimation of the interference waveform. Recently, the Robust Interference Mitigation (RIM) framework was proposed, which leverages results in robust statistics by treating the jamming signal as an outlier. It has the advantage of rejecting jamming signals without detecting or estimating its waveform. In this paper, we extend the framework to situations where the jammer is sparse in some transformed domain other than the time domain. Additionally, we analyse the use of Huber’s non-linearity within RIM and derive its loss of efficiency. We compare its performance to state-of-the-art techniques and to other RIM solutions, with both synthetic and real signals, showing remarkable results.

## 1. Introduction

Global Navigation Satellite System (GNSS) is the technology of choice for most position- and timing-related applications when available [1]. GNSS relies on a constellation of satellites synchronously emitting known signals from which one can compute its position. GNSS encompasses the American GPS, the European Galileo, the Russian Glonass and the Chinese Beidou among others [2,3,4]. The growing dependence on GNSS within critical (and non-critical) infrastructures has posed some concerns about the potential vulnerabilities of GNSS [5]. GNSS signal reception is vulnerable to several forms of Radio Frequency (RF) interference [6] that can significantly degrade receiver performance. For this reason, several approaches have been developed to strengthen GNSS receivers against interference and jamming [5,7,8]. Jamming is a type of intentional interference where a powerful signal is broadcast in the GNSS frequency bands with the ultimate goal of preventing receiver operations. Jamming is a form of Denial of Service (DoS) [9] and can have a significant impact on critical infrastructures relying on GNSS as the location service provider [10].

In the presence of such DoS attacks, GNSS receiver performance can be significantly improved by implementing interference mitigation techniques [7]. These techniques either attempt to cancel the interfering signal or implement processing strategies that are only marginally affected by spurious signal components. Most interference mitigation techniques can be interpreted under the framework of Interference Cancellation (IC) at the receiver. At a glance, IC first detects the jamming signal and then reconstructs its waveform, which is subtracted from the received sample stream to clean the data. Popular IC methods are, for instance, pulse blanking and (adaptive) notch filtering [11,12]. This approach has the drawback of requiring interference detection and estimation, which are two potential points of failure in the chain [13].

In contrast, a novel approach was recently proposed based on the theory of robust statistics [13]. We refer to this approach as RIM, and it has the remarkable advantage of avoiding estimation of the interference waveform and its detection. RIM takes advantage of the sparse nature, in some domain, of interference, given that the desired GNSS signal is generally not sparse. Basically, received signal samples affected by interference can be treated as outliers (after a convenient transformation), and thus their contribution is rejected in a principled manner using robust statistics [14]. The concept was first implemented using a filter to process the jamming signals that were then seen as pulsed interference by the GNSS receiver [15]. The *myriad* non-linearity was derived starting from a Cauchy assumption on the input data corrupted by jamming. The work was extended in [16] using the complex signum non-linearity, where a Laplacian model was considered. Both works substituted the classical Gaussian assumption with a more relaxed assumption on the noise statistics that accounted for heavier tails in the noise probability density function (pdf), typically caused by large outliers in the data. In this paper, we explore the use of Huber’s non-linearity [17] as an alternative to specifying distributions, which is a popular methodology in the field of robust statistics. In practice, this choice entails a piecewise loss function that has higher outlier mitigation capabilities than standard methods based on the Gaussian assumption. Additionally, the RIM concept is extended to interference signals that are sparse in some domain other than the time one. For instance, a Continuous Wave (CW) is an outlier in the frequency domain, where only few samples are affected by the jamming signal. On the other hand, pulsed interference is sparse in the time domain. These and other situations can be formulated in a joint framework, which is one of the contributions of the present article. While this work focuses on time and frequency domain processing, other linear transforms can be used to obtain a sparse signal representation. For example, a Discrete Wavelet Transform (DWT) can be adopted and RIM can be interpreted as a generalization of wavelet excision algorithms [18,19]. The analysis of wavelet-based algorithms is outside the scope of this paper, and it is not considered here.

This work is based on the conference paper in reference [20]. With respect to reference [20], the paper provides several additional contributions. A theoretical analysis of the loss of efficiency caused by Huber’s non-linearity is provided. In particular, a theoretical formula capturing the impact of the decision threshold is derived. The proof of the properties used for the derivation of the loss of efficiency is entirely provided in Appendix A.

The performance of RIM with Huber’s non-linearity is assessed by simulations. Extensive simulations considering two types of jamming signals are provided, namely CW and swept signals. The synthetic simulations presented here are aimed at characterizing the robustness of RIM to different types of interference. This analysis is new and has not been presented before. In addition to this, new experimental results considering tests performed in a large anechoic chamber are presented. The analysis considers both acquisition and tracking stages. The findings obtained complement the results presented in reference [20] that considered narrowband GPS signals collected in a road environment and wideband Galileo E5b signals affected by jamming. Summing up, the results presented here complement both the simulations and the experimental analysis described in reference [20] and provide a complete theoretical analysis of the loss of efficiency entailed by Huber’s non-linearity.

The remainder of the paper is organized as follows. Section 2 presents the GNSS signal model considered in this work, the standard receiver processing based on the maximization of the Cross Ambiguity Function (CAF), and its robust counterpart. Then, Section 3 particularizes the robust CAF concept to the use of Huber’s non-linearity, while Section 4 provides an efficiency analysis of such approach. The proposed methodology is validated with both synthetic and real-world experiments in Section 5 and Section 7, respectively. Section 6 details the experimental configuration for the real data gathering. Finally, the paper concludes with final remarks in Section 8.

## 2. Signal and System Model

The signal collected by the antenna of a GNSS receiver can be modelled as [2]:

(1) $$y\left(t\right)=\sqrt{2C}d\left(t-\tau_{0}\right)c\left(t-\tau_{0}\right)\cos\left(2\pi \left(f_{RF}+f_{0}\right)t+\varphi_{0}\right)+\eta \left(t\right)+i\left(t\right),$$

which is the sum of three terms—the useful signal component, a noise and an interference term. In (1), *C* is the useful signal power, d(·) is a navigation message and c(·) is a pseudo-random code from a family of quasi-orthogonal sequences. The useful signal is delayed by the communication channel that also introduces a Doppler shift, $f_{0}$, with respect to the nominal signal RF, $f_{RF}$. $\tau_{0}$ and $\varphi_{0}$ denote the delay and phase shift introduced by the communication channel. It is noted that a GNSS receiver collects several signals from several satellites. Due to the quasi-orthogonality of the codes, c(·), used to broadcast the different signals, the receiver processes them in an independent way. For this reason, a single useful component is considered in (1).

The noise term, η(t), is a zero-mean Additive White Gaussian Noise (AWGN). The term, i(t), can assume several forms [21,22] depending on the type of interference considered. The receiver amplifies, filters, down-converts and samples y(t), producing the baseband complex sequence

(2) $$y\left[n\right]=\sqrt{C}\tilde{d}\left(nT_{s}-\tau_{0}\right)\tilde{c}\left(nT_{s}-\tau_{0}\right)e^{j2\pi f_{0}nT_{s}+j\varphi_{0}}+\eta_{BB}\left[n\right]+i_{BB}\left[n\right],$$

where *n* is the time index, and square brackets are used to denote discrete time sequences sampled at a frequency of $f_{s}=\frac{1}{T_{s}}$. The subscript “BB” denotes filtered signals down-converted to the baseband. The symbol $\tilde{\cdot }$ indicates the impact of front-end filtering on the useful signal component.

Noise, $\eta_{BB}\left[n\right]$, is modelled as an AWGN with independent and identically distributed (i.i.d.) real and imaginary parts, each with variance, $\sigma^{2}$. A model commonly adopted for $\sigma^{2}$ is

(3) $$\sigma^{2}=N_{0}B_{Rx},$$

where $B_{Rx}$ is the front-end, one-sided bandwidth, and $N_{0}$ is the Power Spectral Density (PSD) of the input noise, η(t). The ratio between the carrier power, *C*, and the noise PSD, $N_{0}$, defines the Carrier-to-Noise power spectral density ratio ($C/N_{0}$).

### 2.1. Standard Processing

The goal of the signal processing stage of a GNSS is to estimate the signal parameters, $\tau_{0}$, $f_{0}$ and $\varphi_{0}$. In standard processing, signal parameters are determined by first computing the CAF, defined as [2]

(4) $$C\left(\tau ,f_{d}\right)=\sum_{n=0}^{N-1}y\left[n\right]c\left(nT_{s}-\tau \right)e^{-j2\pi f_{d}nT_{s}},$$

where *N* is the number of samples used for the computation of the CAF that is integrated over $T_{c}=NT_{s}$, the coherent integration time. The CAF is a bi-dimensional function that depends on τ and $f_{d}$, the locally tested code delay and Doppler frequency. The signal parameters are then computed as

(5) $$\begin{array}{cc} \left\{\hat{\tau },{\hat{f}}_{d}\right\} & =\arg\;\max_{\tau ,f_{d}}\left|C\left(\tau ,f_{d}\right)\right|\\ \hat{\varphi } & =∠C(\hat{\tau },{\hat{f}}_{d}) \end{array}.$$

The maximization process described by (5) is implemented by acquisition and tracking algorithms that employ a bank of correlators to compute and maximize the CAF. The correlators are also used to estimate the $C/N_{0}$ that is thus determined post-correlation. The interference term, $i_{BB}\left[n\right]$, can significantly distort the CAF, preventing proper receiver operations. In this way, the estimated $C/N_{0}$ is strongly influenced by the interference term, $i_{BB}\left[n\right]$, and by the interference mitigation strategies implemented by the receiver. In this respect, the estimated $C/N_{0}$ can be used to quantify the effectiveness of interference mitigation approaches. Pre-correlation mitigation techniques, such as those based on Huber’s non-linearity, are applied before the computation of (4) on the digital samples, y[n], which are, at first, pre-processed to reduce the impact of $i_{BB}\left[n\right]$.

### 2.2. Robust Interference Mitigation

**Figure 1:**
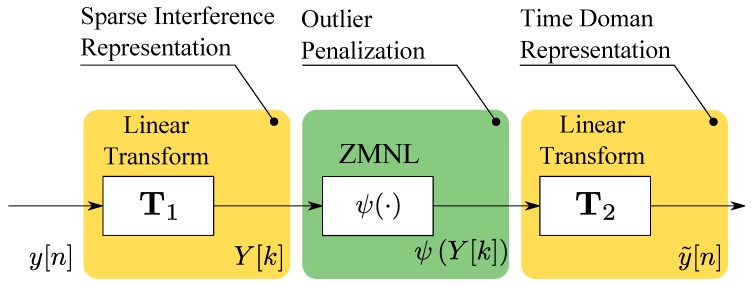
Three main processing steps required to implement RIM that is a pre-correlation mitigation technique.

RIM is a pre-correlation technique, i.e., applied directly to the samples, y[n], before correlation, which can be implemented according to the three steps depicted in Figure 1 [20,23]. A linear tranform, $T_{1}$, is, at first, used to project the jamming component into a domain where it admits a sparse representation, i.e., it affects a limited number of samples. For example, a low-pass filter can be used to make the jamming component appear as a sequence of time pulses [15,24]. This approach is effective for instantaneously narrowband jamming signals that sweep a large frequency band. When filtered, these signals result in a sequence of pulses. Transform Domain (TD) processing was suggested by [23], where the DFT was used to project the samples, y[n], into the frequency domain where $i_{BB}\left[n\right]$ admitted a sparse representation. Transform $T_{1}$ produces the TD samples

(6) $$Y\left[k\right]=T_{1}\left(y\left[n\right]\right).$$

The change of index, from *n* to *k*, is a notational convention adopted to indicate that the input samples, y[n], have been brought to a different representation domain. When $T_{1}$ is the identity operator, the change of index used for the enumeration of the TD samples is no longer required.

Following $T_{1}$, a Zero Memory Non-Linearities (ZMNL) is used to reduce the impact of outliers in the TD. The complex signum and myriad non-linearities were considered in reference [15,24]. In this paper, Huber’s non-linearity [17,25] is analysed. While this ZMNL is widely used in robust statistics, its application to complex samples for interference mitigation is new, and it is one of the main contributions of this paper. A generic non-linearity is denoted here as ψ(·) and produces the samples

(7) $$Y_{\psi }\left[k\right]=\psi \left(Y\left[k\right]\right)\;.$$

Finally, a second linear transform, $T_{2}$, is applied to the samples, $Y_{\psi }\left[k\right]$, to obtain new filtered time domain samples. $T_{2}$ inverts the effects of $T_{1}$ and brings back the samples from the TD to the time domain. In time domain processing, $T_{2}$ is simply the identity. The output of $T_{2}$ is denoted here as

(8) $$\tilde{y}\left[n\right]=T_{2}\left(Y_{\psi }\left[k\right]\right).$$

As a consequence, two special cases of RIM can be identified:

- *Time domain processing*: when $T_{1}$ is a low pass-filter and $T_{2}$ is the identity operator. In narrowband receivers, low-pass filtering is not needed. and $T_{1}$ can be replaced by the identity operator,
- *TD processing*: $T_{1}$ and $T_{2}$ are inverse operators and
  (9) $$T_{1}\circ T_{2}=I,$$

where I is the identity operator. If $T_{1}$ and $T_{2}$ can be represented as invertible square matrices, they are orthogonal projection operators that change the signal representation basis. In this paper, we consider only orthonormal transformations that preserve power relationships.

RIM aims to reduce the impact of $i_{BB}\left[n\right]$ on the final cleaned samples, $\tilde{y}\left[n\right]$, which are used for the computation of the robust CAF [15]:

(10) $$C_{\psi }\left(\tau ,f_{d}\right)=\sum_{n=0}^{N-1}\tilde{y}\left[n\right]c\left(nT_{s}-\tau \right)e^{-j2\pi f_{d}nT_{s}}.$$

In this respect, after RIM, a standard receiver architecture can be adopted where $\tilde{y}\left[n\right]$ are used instead of the original samples, y[n].

## 3. Huber’s Non-Linearity

As mentioned in the previous section, the focus of this paper is Huber’s non-linearity, defined as [26]

(11) $$\psi_{H}\left(Y\left[k\right]\right)=\left\{\begin{array}{cc} Y[k] & \;\mathrm{for}\;|Y\left[k\right]|\leq T_{h}\\ T_{h}\;\mathrm{csign}\left(Y\left[k\right]\right) & \;\mathrm{for}\;|Y\left[k\right]|>T_{h} \end{array}.\right.$$

where $T_{h}$ is a decision threshold. The ZMNL implements outlier detection on the TD samples, Y[k]; if their amplitude is lower than $T_{h}$ they are considered inliers and they are left unchanged. Otherwise, their amplitude is clipped to $T_{h}$. The phase of the samples, Y[k], is preserved through the complex signum operator, defined as [27]

(12) $$\mathrm{csign}\left(Y\left[k\right]\right)=\left\{\begin{array}{cc} \frac{Y[k]}{\left|Y[k]\right|} & \;\mathrm{for}\;Y[k]\neq 0\\ 0 & \;\mathrm{for}\;Y[k]=0 \end{array}\right..$$

ZMNL (11) is the derivative of Huber’s penalty function that was originally designed to introduce robustness to the standard Least Squares (LS) estimation [17,25]. The original derivations from Huber considered real input samples [17,25]. We extended the definition of Huber’s ZMNL to complex samples using the phase decoupling approach [20,28].

**Figure 2:**
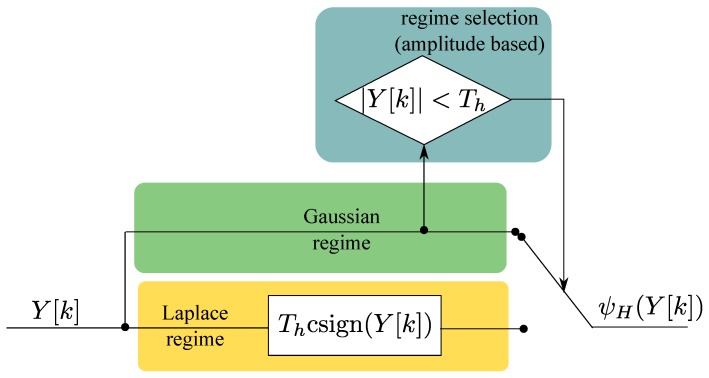
Processing implemented by Huber’s non-linearity.

The Complex signum non-linearity arises when modelling the joint pdf of $i_{BB}\left[n\right]$ and $\eta_{BB}\left[n\right]$ as Laplace [24]. It can be seen that when $|Y\left[k\right]|>T_{h}$, the applied non-linearity is equivalent to the one resulting from the Laplace assumption [16]. The processing implemented by Huber’s non-linearity can thus can be interpreted as a switch between two regimes: the Gaussian and the Laplace ones. This fact is highlighted in Figure 2 which shows the two branches of Huber’s non-linearity.

The amplitude of the input samples, Y[k], determines the switch between Gaussian and Laplace regimes. The decision threshold can be set according to different criteria. A possibility is to consider the passage between the two regimes as a test on the amplitude of the input samples, Y[k]. In this respect, (11) can be considered to be a form of interference detection: if the input samples have a magnitude larger than $T_{h}$, then interference is detected and the complex signum non-linearity is applied. The decision threshold, $T_{h}$, can be set in order to guarantee a constant false alarm rate [29]:

(13) $$P_{fa}\left(T_{h}\right)=P(|Y\left[k\right]|>T_{h}\left|H_{0}\right)$$

where $H_{0}$ denotes the null hypothesis consisting of the absence of interference. Under $H_{0}$, Y[k] follows a complex circularly symmetric Gaussian distribution, and its amplitude follows a Rayleigh distribution.

In Section 5, we set $T_{h}$ according to the empirical value determined by Huber [30] for real random variables, whereas we performed a parametric analysis in Section 7 where different threshold settings are considered. In all cases, the threshold, $T_{h}$, should be obtained by scaling the noise standard deviation, σ. This fact is better discussed in the next section where the loss of efficiency is analysed. This loss only depends on the normalized threshold, $\frac{T_{h}}{\sigma }$.

## 4. Efficiency Analysis

In this section, the loss of efficiency caused by Huber’s non-linearity is theoretically evaluated. The loss of efficiency is the performance degradation caused by the non-linearity in the absence of interference, and it is measured with respect to the optimal Gaussian estimator, i.e., with respect to standard GNSS processing [17]. The loss of efficiency is given by

(14) $$L_{0}\left(T_{h}\right)=\frac{{\mathrm{SNR}}_{out}^{\psi }}{{\mathrm{SNR}}_{out}}$$

and is the ratio between the post-correlation Signal-to-Noise Ratio (SNRs) obtained in the absence of interference, i.e., for $i_{BB}\left[n\right]=0$. The post-correlation SNR measures the quality of the CAF, and it is defined as [31,32]

(15) $${\mathrm{SNR}}_{out}=\max_{\tau ,f_{d}}\frac{{\left|E\left\{C(\tau ,f_{d})\right\}\right|}^{2}}{\frac{1}{2}\mathrm{Var}\left\{C(\tau ,f_{d})\right\}}.$$

When Huber’s non-linearity is applied, the standard CAF, $C(\tau ,f_{d})$, is replaced by its robust counterpart, defined in Section 2.2. Thus, ${\mathrm{SNR}}_{out}^{\psi }$ is obtained according to definition (15), where $C_{\psi }\left(\tau ,f_{d}\right)$ replaces the standard CAF, $C(\tau ,f_{d})$.

In order to evaluate ${\mathrm{SNR}}_{out}^{\psi }$, it is necessary to determine the first two moments of the robust CAF, $C_{\psi }\left(\tau ,f_{d}\right)$. In particular,

(16) $$E\left\{C_{\psi }\left(\tau ,f_{d}\right)\right\}=\sum_{n=0}^{N-1}E\left\{\tilde{y}\left[n\right]\right\}c\left(nT_{s}-\tau \right)e^{-j2\pi f_{d}nT_{s}}.$$

The first moment of the robust CAF is proportional to $E\left\{\tilde{y}\left[n\right]\right\}$, which depends on the transformations, $T_{1}$ and $T_{2}$. It is possible to show [23] that when $T_{1}$ and $T_{2}$ are orthonormal transformations, the statistical properties of the input samples, y[n], are preserved. Orthonormal transformations preserve the Gaussian nature of the noise affecting the input samples and preserve their variance. Recall that, for the loss of efficiency analysis, we are interested in a nominal, no-jamming situation where the Gaussian assumption is plausible. In addition to this, no correlation is introduced by orthonormal transformations among the input samples. For this reason, the analysis provided in the following is conducted on the input samples, y[n], and $T_{1}$ and $T_{2}$ are set equal to the identity. The results obtained are, however, general and apply to orthonormal transformations, such as the DFT and Inverse DFT (IDFT). The case of non-orthonormal transformations is outside the scope of this paper.

When $T_{1}=T_{2}=I$, $\tilde{y}\left[n\right]=\psi_{H}\left(y\left[n\right]\right)$ and

(17) $$E\left\{\psi_{H}\left(y\left[n\right]\right)\right\}=E\left\{y[n]\right\}\left[1-e^{-\frac{T_{h}^{2}}{2\sigma^{2}}}+\frac{\sqrt{\pi }}{2}\frac{T_{h}}{\sqrt{2}\sigma }\mathrm{erfc}\left(\frac{T_{h}}{\sqrt{2}\sigma }\right)\right]=E\left\{y[n]\right\}A\left(\frac{T_{h}}{\sqrt{2}\sigma }\right)$$

with

(18) $$A\left(\frac{T_{h}}{\sqrt{2}\sigma }\right)=1-e^{-\frac{T_{h}^{2}}{2\sigma^{2}}}+\frac{\sqrt{\pi }}{2}\frac{T_{h}}{\sqrt{2}\sigma }erfc\left(\frac{T_{h}}{\sqrt{2}\sigma }\right).$$

Function erfc(·) in (17) and (18) is the complementary error function [33]. The proof of (17) is provided in Appendix A and shows that, on average, the samples processed with Huber’s non-linearity are proportional to the input samples, y[n]. On average, the non-linearity causes a scaling that is proportional to (18). The scaling only depends on the normalized threshold, $\frac{T_{h}}{\sqrt{2}\sigma }$. Combining (16) with (17), it also follows that the robust CAF is, on average, proportional to the standard CAF:

(19) $$E\left\{C_{\psi }\left(\tau ,f_{d}\right)\right\}=A\left(\frac{T_{h}}{\sqrt{2}\sigma }\right)E\left\{C(\tau ,f_{d})\right\}.$$

This result implies that, on average, the CAF is only scaled by Huber’s non-linearity. For this reason, no biases are introduced on the signal parameters that are estimated as the values corresponding to the maximum value of the CAF.

The variance of the robust CAF can be computed using a similar approach and exploiting the independence of the noise samples. In more detail,

(20) $$\begin{array}{cc} Var\left\{C_{\psi }\left(\tau ,f_{d}\right)\right\} & =Var\left\{\sum_{n=0}^{N-1}\tilde{y}\left[n\right]c\left(nT_{s}-\tau \right)e^{-j2\pi f_{d}nT_{s}}\right\}\\ & =\sum_{n=0}^{N-1}Var\left\{\tilde{y}\left[n\right]\right\}=\sum_{n=0}^{N-1}Var\left\{\psi_{H}\left(y\left[n\right]\right)\right\}. \end{array}$$

The absence of cross-correlation terms in (20) is due to the independence of the input noise samples and to the fact that $\psi_{H}\left(\cdot \right)$ does not introduce memory. The variance of $\psi_{H}\left(y\left[n\right]\right)$ is computed in Appendix A for weak signal conditions and is given by

(21) $$\mathrm{Var}\left\{\psi_{H}\left(y\left[n\right]\right)\right\}=2\sigma^{2}\left[1-e^{-\frac{T_{h}^{2}}{2\sigma^{2}}}\right]=\mathrm{Var}\left\{y[n]\right\}\left[1-e^{-\frac{T_{h}^{2}}{2\sigma^{2}}}\right].$$

Note that the weak signal condition is very plausible in the context of GNSS signals, which are known to be received with very low power levels compared to the noise floor [2]. By combining (20) and (21), it follows that

(22) $$\mathrm{Var}\left\{C_{\psi }\left(\tau ,f_{d}\right)\right\}=\mathrm{Var}\left\{C(\tau ,f_{d})\right\}\left[1-e^{-\frac{T_{h}^{2}}{2\sigma^{2}}}\right]$$

and

(23) $${\mathrm{SNR}}_{out}^{\psi }=\frac{A^{2}\left(\frac{T_{h}}{\sqrt{2}\sigma }\right)}{1-e^{-\frac{T_{h}^{2}}{2\sigma^{2}}}}\max_{\tau ,f_{d}}\frac{{\left|E\left\{C(\tau ,f_{d})\right\}\right|}^{2}}{\frac{1}{2}\mathrm{Var}\left\{C(\tau ,f_{d})\right\}}=L_{0}\left(T_{h}\right){\mathrm{SNR}}_{out}.$$

From the above results, it follows that the loss of efficiency is given by

(24) $$L_{0}\left(T_{h}\right)=\frac{{\left[1-e^{-\frac{T_{h}^{2}}{2\sigma^{2}}}+\frac{T_{h}}{\sqrt{2}\sigma }\frac{\sqrt{\pi }}{2}\mathrm{erfc}\left(\frac{T_{h}}{\sqrt{2}\sigma }\right)\right]}^{2}}{1-e^{-\frac{T_{h}^{2}}{2\sigma^{2}}}}.$$

This result was obtained under weak signal conditions, and it is valid for orthonormal transformations. In [23], the validity of (24) was evaluated through simulations considering time domain processing, i.e., with $T_{1}=T_{2}=I$. In the following section, the validity of (24) is verified through simulations for frequency domain processing. $T_{1}$ implements a DFT of size *N*, while $T_{2}$ implements the inverse transform, the IDFT.

### Validation through Simulations

In order to support the validity of (24) for orthonormal transformations, Monte Carlo simulations were used to compute the post-correlation SNRs and estimate the loss of efficiency. Frequency domain processing was implemented where the DFT and its inverse were used as linear transformations, $T_{1}$ and $T_{2}$. The two transforms were normalized in order to preserve norms and signal energy. Notice that in reference [20], the special case of $T_{1}=T_{2}=I$ was considered.

The parameters of the signal used for the purpose of showing the loss of efficiency can be consulted in Table 1. Particularly, GPS L1 Coarse Acquisition (C/A) signals were generated according to model (2) with $i_{BB}\left[n\right]=0$. Simulated signals were used to compute several realizations of the CAF which, in turn, were used to estimate the post-correlation SNR, defined in (15). Simulated signals were processed and integrated using a local code and carrier with the delay and Doppler frequency matching the parameters of the simulated components. When the parameters of the local signal matched those of the incoming samples, the CAF is maximized. The CAF was computed with and without RIM, and both ${\mathrm{SNR}}_{out}$ and ${\mathrm{SNR}}_{out}^{\psi }$ were evaluated. Finally, loss (24) was determined as a function of $T_{h}$.

The asymptotic analysis of (24) showed that, for large values of $T_{h}$,

(25) $$L_{0}\left(T_{h}\right)\to 1,\;\mathrm{for}\;T_{h}\to \infty .$$

This result is consistent with the fact that for large values of $T_{h}$, $\psi_{H}\left(\cdot \right)$ becomes the identity and no processing is applied to the TD samples. If the threshold is too large, the presence of interference is never detected and no processing is applied. In this way, no loss is introduced. For small values of $T_{h}$, $\psi_{H}\left(\cdot \right)$ degenerates to the complex signum non-linearity [24] and

(26) $$L_{0}\left(T_{h}\right)\to \frac{\pi }{4}=0.7854\left(-1.049\;dB\right),\;\mathrm{for}\;T_{h}\to 0.$$

**Figure 3:**
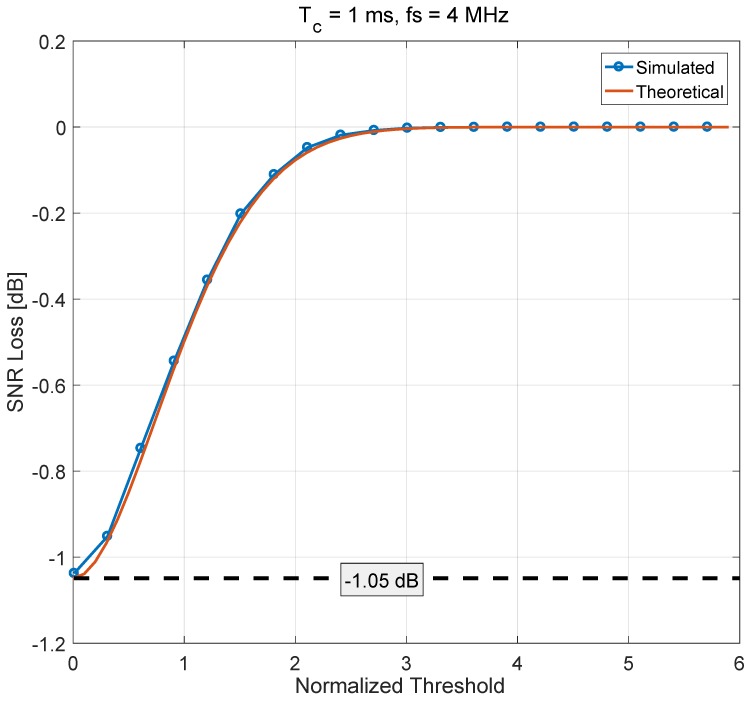
Loss of efficiency as a function of the normalized threshold, $\frac{T_{h}}{\sigma }$: comparison between theoretical and simulation results. Results obtained considering frequency domain processing.

A good agreement between simulations and theoretical results is observed in Figure 3. The loss, which is provided in dB scale, monotonically increases as a function of the normalized threshold, $T_{h}/\sigma$, from a minimum of −1.049 dB to a maximum of 0 dB. When the loss is above 3σ, the effect of Huber’s non-linearity is negligible, and the loss approaches unity. The analysis confirms the validity of the loss derived in (24), where the loss of efficiency under nominal conditions is relatively small.

The loss of efficiency in the absence of jamming is expected to be the same for both time and frequency domain processing. This fact is confirmed by the curve obtained by simulations, and is shown in Figure 3. The loss of efficiency determined using DFT/IDFT transformations is the same as that shown in [20] where time domain processing was considered.

## 5. Simulation Analysis

RIM was validated using a synthetically controlled interference which allowed for adjustment of its parameters. The interference was injected on a real capture of a GPS L1 C/A signal. The signal was captured with a Universal Software Radio Peripheral (USRP) front-end, with a sampling frequency of $f_{s}=4$ MHz and a front-end bandwidth of 2 MHz. The length of the capture was about 10 s. In the absence of interference and using a standard receiver approach that does not implement RIM, the total number of available satellites was 5, with an average $C/N_{0}$ of 46 dB-Hz. The Phase Lock Loop (PLL) and Delay Lock Loop (DLL) bandwidths were 10 Hz and 2 Hz, respectively.

Two kinds of jamming signals were injected into the signal capture. On one hand, a CW interference with a fixed frequency at 1 kHz with respect to the central frequency (e.g., the baseband in these experiments) was used, and this is mathematically defined as

(27) $$i_{\mathrm{CW}}\left[n\right]=A_{J}e^{j2\pi f_{J}nT_{s}}\;,$$

where $A_{J}$ is the amplitude of the jamming signal, and $f_{J}=1$ kHz is the jamming frequency. On the other hand, the second interference generated was a Saw-Tooth (ST) signal with frequency sweeping from 0 to 4 MHz, repeated periodically every 10 μs. The ST is defined as

(28) $$i_{\mathrm{ST}}\left[n\right]=A_{J}e^{j2\pi T_{s}\sum_{j=0}^{n}f_{J}\left[j\right]}\;,$$

where $f_{J}\left[n\right]$ is the instantaneous frequency.

The performance of the different robust methods was compared with adaptive notch filtering and a standard receiver implementing (4) with no anti-jamming. The assessment of RIM methods included the one proposed in this article, based on Huber’s non-linearity, but also those based on the Laplace and Cauchy assumptions. In the figures, we denote the latter non-linearities by *complex signum* [16] and *myriad* [15], respectively. We use the PLL discriminator variance for satellite 11 in the dataset versus Jamming to Noise power ratio (J/N) as a metric of tracking quality. Huber’s threshold was set to $T_{h}=1.345\sigma$, which is known to be optimal for the real-valued signal case [30]. The myriad non-linearity used in this paper was studied in [15] and takes the form

(29) $$\psi_{M}\left(Y\left[k\right]\right)=\frac{K}{K+{\left|Y\left[k\right]\right|}^{2}}$$

where $K=6\sigma^{2}$ was selected for the processing performed. This value was selected according to the findings detailed in [15]. Note that all of the robust methods considered here operate in the frequency domain. The J/N was determined by the total noise variance, $2\sigma^{2}$, and the amplitude of the jamming signal:

(30) $$\frac{J}{N}=\frac{A_{J}^{2}}{2\sigma^{2}}.$$

In our approach, we first estimated the noise variance using the clean samples collected in the absence of interference. The J/N was then fixed, and $A_{J}$ was determined by inverting (30).

**Figure 4:**
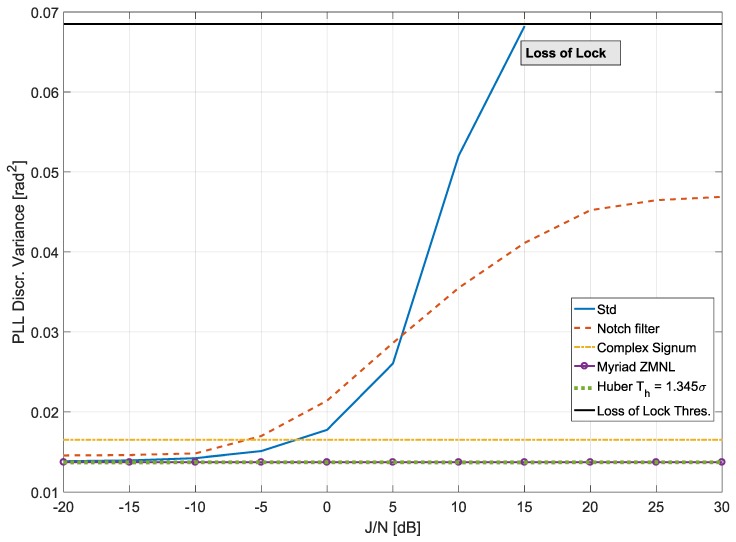
Variance of the PLL discriminator versus J/N of a CW interference. Comparison of standard (no anti-jamming) processing, adaptive notch filtering, complex signum ZMNL, myriad ZMNL and Huber’s ZMNL.

**Figure 5:**
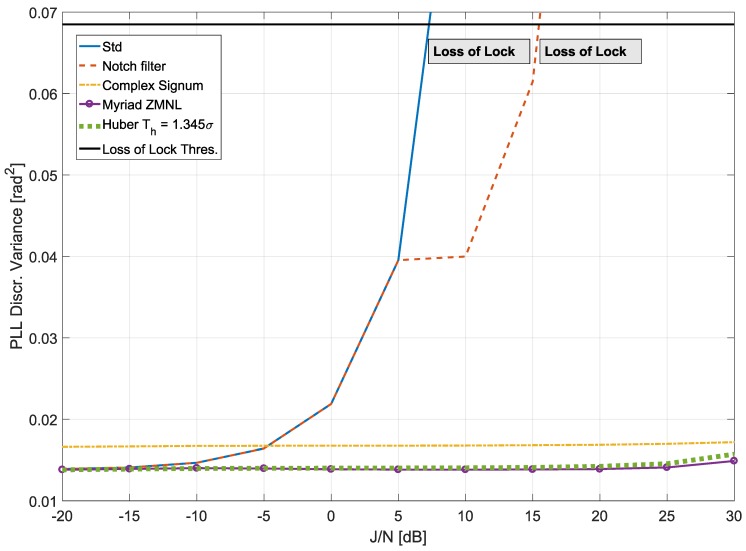
Variance of the PLL discriminator versus the J/N of a swept interference. Comparison of standard (no anti-jamming) processing, adaptive notch filtering, complex signum ZMNL, myriad ZMNL and Huber’s ZMNL.

Given the superior performance of DLL under high noise conditions, we focused the study on evaluating the impact on the PLL. The results are provided in Figure 4 and Figure 5 for the two jamming signals being tested. The adaptive notch filter used in this paper was the Infinite Impulse Response (IIR) one-pole filter analysed in [34,35]. The transfer function of the filter was as follows:

(31) $$H\left(Z\right)=\frac{1-z_{0}\left[n\right]z^{-1}}{1-k_{\alpha }z_{0}\left[n\right]z^{-1}}\;,$$

where $z_{0}\left[n\right]$ is the filter zero, and $k_{\alpha }$ is the pole contraction factor that controls the width of the notch. Note that $z_{0}$ determines the current notch centre frequency, $\Phi \left(nT_{s}\right)=\frac{f_{s}}{2\pi }∠z_{0}\left[n\right]$. Ideally, this frequency should correspond to the interference centre frequency. The adaptive notch filter is indeed designed to adjust $z_{0}\left[n\right]$ in order to track the jamming frequency [34]. In this paper, $k_{\alpha }$ was chosen as 0.7 for the ST jamming signal and 0.9 for the CW jamming signal.

Figure 4 shows the results obtained when considering the case of CW interference. The black line represents the threshold for the PLL loss of lock, which occurs when the standard deviation of the discriminator is larger than $15^{\circ }$ (corresponding to a variance of 0.068 rad^2^) [2]. For low J/N conditions, Huber’s non-linearity and myriad ZMNL performed similarly to standard processing, which is optimal in the absence of interference. As the J/N increases, the variance of the discriminator output, obtained in the absence of mitigation, progressively increased, leading to a loss of lock for a J/N of about 15 dB. At low J/N, the adaptive notch filter introduced a small variance degradation. This is due to the fact that the jamming signal was too weak, and the notch filter was unable to estimate its frequency. Note that the adaptive notch filter removesd a portion of the signal spectrum. In this respect, a part of the useful signal component was also removed. When the CW interference was characterized by a J/N greater than 5 dB, the adaptive notch filter was able to track it and effectively remove it. For J/N greater than 20 dB, the notch filter effectively estimated the interference frequency, and the variance of the discriminator output remained almost constant. The notch filter allowed the receiver to operate for larger J/N values than standard processing. In this respect, the receiver did not lose lock under the tested conditions. Robust approaches operate differently from the notch filter and, in particular, they do not need to estimate the frequency of the CW interference. In the frequency domain, the CW has a sparse support, and only a very limited number of frequency samples is affected. The support of the CW signal in the frequency domain does not depend on the J/N, and for this reason, the variance of the discriminator output is constant. This effect is clearly visible in Figure 4.

Figure 5 shows the tracking results obtained in the case of a ST jamming signal. For low J/N conditions, the adaptive notch filter performs slightly better with respect to the CW jamming signal case. As the J/N increased, the variance of the discriminator output obtained in the absence of mitigation progressively increased, leading to loss of lock for J/N of about 8 dB. A similar trend was observed for the adaptive notch filter, which was not able to track the variation in the jamming signal. The residual interference components not removed by the notch filter caused a progressive increase in the discriminator variance.

From Figure 5, it is possible to observe that RIM methods provided almost invariant performance in the J/N range analysed. Besides, the discriminator variance was always kept below the loss of lock threshold. RIM allowed receiver operations for all of the conditions tested

## 6. Experimental Setup

The effectiveness of Huber’s non-linearity for RIM was experimentally evaluated using the data collected during the anechoic chamber experiment described in reference [22,36]. These data had not been used before for the analysis of Huber’s non-linearity and thus they provide results complementary to those obtained in reference [20], which considered different experiments and signals.

**Figure 6:**
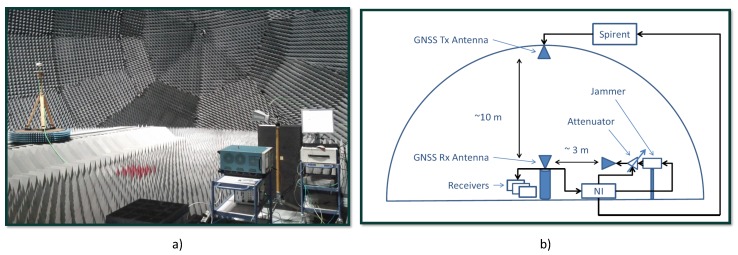
Anechoic chamber experiment involving hardware-simulated GPS and Galileo signals. (**a**) View of the anechoic chamber. (**b**) Schematic representation of the experimental setup.

As described in [22], the experiments were performed inside a large anechoic chamber available at the Joint Research Centre (JRC) in Ispra, Italy. The tests involved an in-car jammer and a Spirent GSS8800 GNSS hardware simulator. The jammer broadcast a ST signal able to span a frequency range of about 12 MHz in about 9 μs. Its power was varied through a programmable attenuator placed between the jammer antenna connector and the transmitting antenna. The attenuator was used to create a variable jamming power profile—at the beginning of the experiment, the attenuator was set to its maximum value with an attenuation equal to 81 dB. The attenuation was progressively reduced in 2 dB decrements to a minimum value of 45 dB. Finally, the attenuation was again increased in 2 dB increments to its maximum value. A view of the anechoic chamber where the experiment was conducted is provided in Figure 6a, whereas Figure 6b provides a schematic representation of the experimental setup.

The data collected in the anechoic chamber were used to test different interference mitigation techniques [22,36] that could be directly compared with Huber’s non-linearity analysed here. The attenuation profile, the corresponding J/N and additional information on the setup can be found in [22,36], and thus, they are not repeated here.

The Spirent GSS8800 simulator was used to generate GPS L1 C/A and Galileo E1 signals that were recorded along with the jamming signal. A high-fidelity National Instruments (NI) RF signal analyser (NI PXI-5663) was used for the data collection. The parameters adopted for data recording are provided in Table 2.

## 7. Experimental Results

In this section, the experimental results obtained using the data collected according to the setup described in Section 6 are analysed. Both GPS L1 C/A and Galileo E1c signals were considered. More specifically, the raw In-phase Quadrature (I/Q) data collected with the NI PXI-5663 front-end were processed with a custom Matlab software receiver, implementing RIM and using Huber’s non-linearity. The receiver adopted a standard architecture based on standard acquisition and tracking [2]. The parameters adopted for the processing of the GPS and Galileo signals are provided in Table 3.

RIM was implemented as a pre-correlation technique on the input samples before further processing. Acquisition and tracking were analysed separately. Acquisition performance was assessed at first by comparing the CAFs obtained in the presence of jamming with and without RIM. Tracking was analysed by considering the effective $C/N_{0}$ [31,32] estimated using the correlator outputs obtained using standard DLLs and PLLs. During tracking, the receiver continuously estimates the signal $C/N_{0}$, which is determined post-correlation, and it is thus proportional to the post-correlation SNR introduced in Section 4. In particular, the $C/N_{0}$ is obtained by first estimating (15) and scaling it with respect to the coherent integration time, $T_{c}$:

(32) $$\frac{C}{N_{0}}=\frac{1}{2T_{c}}{\mathrm{SNR}}_{out}.$$

For this reason, the effective $C/N_{0}$ reflects the impact of interference, which is perceived for wideband jamming signals as a noise increase, and interference mitigation. In this respect, many research papers [36,37,38,39] express the performance improvements obtained using interference mitigation in terms of $C/N_{0}$ gain. The $C/N_{0}$ estimated by the receiver quantifies the ability of a specific technique to mitigate the impact of jamming, and it is used in the following text for performance evaluation.

The $C/N_{0}$ values obtained considering different mitigation techniques are compared in the following in order to evaluate the performance of Huber’s non-linearity. Both time domain and frequency domain processing are considered for both GPS and Galileo signals. The Galileo E1c signal is a pilot channel. For this reason, a pure PLL with a four-quadrant arctangent is used after achieving secondary code synchronization.

### 7.1. Time Domain Processing

Time domain processing was implemented using a low-pass filter to make the jamming signal appear as a sequence of pulses [36]. The low-pass filter materialized the transformation, $T_{1}$, while $T_{2}$ was set equal to the identity. The characteristics of the low-pass filter were selected according to the properties of the signal to be processed. In this respect, the spectrum of the GPS L1 C/A modulation was narrower than that of the Galileo E1c signal. For this reason, it was possible to use a narrower filter for the processing of the GPS L1 C/A signal.

**Figure 7:**
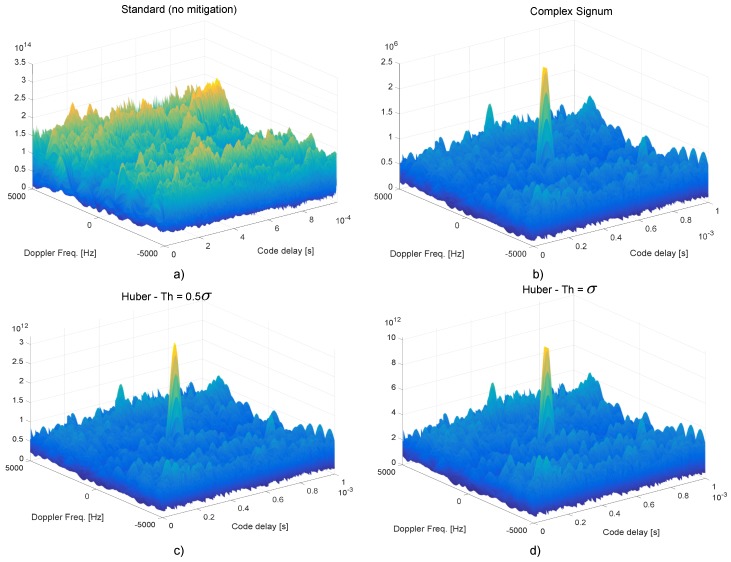
CAFs obtained using different time domain processing strategies and for high jamming levels. The data used for the CAF computation are from the anechoic chamber experiment and were collected after 1000 s from the start of the test. (**a**) Standard processing without mitigation. (**b**) Complex signum non-linearity. (**c**) Huber’s non-linearity with $T_{h}=0.5\sigma$. (**d**) Huber’s non-linearity with $T_{h}=\sigma$.

The CAFs obtained using time domain RIM for the GPS L1 C/A signal are shown in Figure 7, which considers the standard case without mitigation, the usage of the complex signum non-linearity and two configurations adopting Huber’s ZMNL with $T_{h}=0.5\sigma$ and $T_{h}=\sigma$, respectively. The CAFs were computed using the dataset collected during the anechoic chamber experiment described in Section 6. In particular, the data used for the CAF computation were extracted from the dataset after 1000 s from the beginning of the experiment. At this point, a J/N=12 dB was experienced. A coherent integration time, $T_{c}=1$ ms, and 10 non-coherent integrations were used for the CAF computation. As shown in Section 5, standard processing and notch filtering were not able to maintain the signal lock for this J/N level. Similarly, for this J/N level, standard processing was unable to reveal the signal presence. This fact clearly emerges from the CAF shown in Figure 7a—no clear signal peak emerges from the CAF noise floor when interference mitigation is not implemented. When RIM is implemented, in this case a low-pass filter with a 1 MHz cut-off frequency is used, a clear signal peak can be identified in the CAFs. The CAFs shown in Figure 7b–d have similar shapes and clearly reveal the presence of the useful signal. Similar results were obtained from the three non-linearities considered in Figure 7. While the CAF analysis provided in Figure 7 is qualitative in nature, it clearly shows the benefits of RIM at the acquisition stage. In the following section, we will focus on tracking performance, evaluated using the effective $C/N_{0}$ that was estimated post-correlation. This metric allows a more qualitative analysis of RIM and its ability to remove interference terms when computing the correlator outputs. This allows a better comparison between different non-linearities.

**Figure 8:**
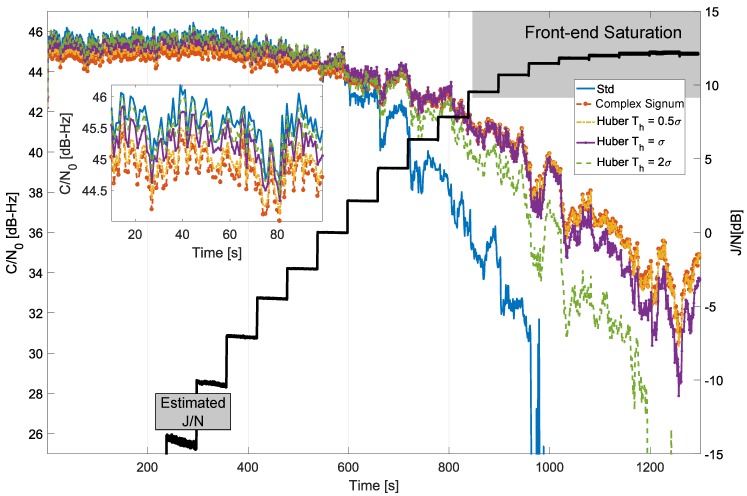
Comparison of $C/N_{0}$ values obtained considering different time domain processing schemes. Anechoic chamber test with GPS signals.

The results obtained for the time domain processing of the GPS L1 C/A signal are provided in Figure 8, which analyses the $C/N_{0}$ values obtained by considering different parameters for Huber’s non-linearity, compared with standard processing and with the complex signum non-linearity. In this case, a low-pass filter with a 1 MHz cut-off frequency was used to protect the main lobe of the GPS L1 C/A modulation. When standard processing was adopted, i.e., without interference mitigation, the receiver was unable to maintain the signal lock for high jamming power levels. In Figure 8, standard tracking lost lock after about 960 s when the J/N was about 12 dB, and front-end saturation started to occur [22]. Figure 8 also provides the J/N estimated from the collected samples. In particular, the noise power, *N*, was, at first, estimated using the samples collected at the beginning of the experiment when the jamming power, *J*, could be neglected. The noise power was considered to be constant throughout the whole duration of the experiment, and the remaining samples were then used to estimate the total received power given by the sum of *J* and *N*. From the total received power and *N*, the J/N shown in Figure 8 was finally estimated. After about 900 s from the start of the test, a significant reduction in the steps describing the J/N behaviour was observed. This phenomenon is due to front-end saturation. Indeed the jamming signal was so powerful that it was clipped by the Analog-to-Digital Converter (ADC) quantization function. This led to an apparent reduction in the received jamming power. However, front-end saturation generates harmonics that spread the jamming power on different frequencies reducing the effectiveness of RIM. This phenomenon is equivalent to increasing the number of outliers that affect the received samples. The estimated J/N is reported also in the following figures to provide a better evaluation of the improvements led by RIM. Despite the impact of saturation, RIM provided significant protection against jamming, and the receiver was able to maintain signal lock for all the configurations tested except for the case with $T_{h}=2\sigma$.

When the decision threshold was too high, as for $T_{h}=2\sigma$, significant levels of interference passed through undetected and thus were unmitigated. In these cases, the processed samples, $\tilde{y}\left[n\right]$, were still affected by interference that compromised the receiver performance. This fact clearly emerges in Figure 8 for the $T_{h}=2\sigma$ case—while Huber’s non-linearity was able to improve receiver performance for medium-to-high levels of interference, the large detection threshold, $T_{h}=2\sigma$, did not allow the receiver to maintain signal lock for the whole duration of the test. The receiver performance further increased by reducing $T_{h}$. This fact clearly emerges from Figure 8—for $T_{h}=0.5\sigma$ and $T_{h}=\sigma$, the receiver was able to maintain lock for the whole duration of the test. Small values of $T_{h}$ increased the robustness to jamming. On the other hand, the efficiency of RIM, i.e., the receiver performance in the absence of interference, decreased with $T_{h}$. This fact was discussed from a theoretical point of view in Section 4, and it is further analysed in the box in the bottom left part of Figure 8. This box shows the $C/N_{0}$ values obtained at the beginning of the experiment when jamming levels can be neglected. As expected, standard processing provided the best performance as no loss of efficiency occurred. The $C/N_{0}$ loss increased with the reduction of $T_{h}$. For $T_{h}=2\sigma$, $C/N_{0}$ values close to those obtained for standard processing case were observed. For small threshold values, $C/N_{0}$ losses close to 1 dB were observed. These results are in agreement with the theoretical results discussed in Section 4. Figure 8 also provides the $C/N_{0}$ obtained considering the complex signum non-linearity. As discussed in Section 4, this ZMNL can be considered to be the limit case of Huber’s non-linearity for a vanishing small threshold, i.e., when all the input samples are considered to be affected by interference. In this case, only the phase information is preserved. As expected, the complex signum ZMNL provided the best performance in terms of interference mitigation, but, at the same time, it had the highest loss of efficiency. The threshold of Huber’s non-linearity allows one to select the best compromise between loss of efficiency and robustness to interference. These results are in agreement with the findings presented in reference [20].

**Figure 9:**
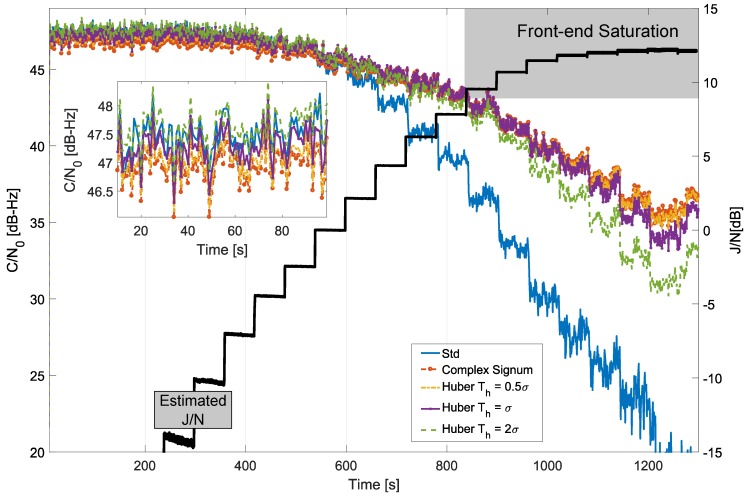
Comparison of $C/N_{0}$ values obtained considering different time domain processing schemes. Anechoic chamber test with Galileo E1c signals.

The $C/N_{0}$ values obtained for the processing of the Galileo E1c signal are provided in Figure 9. In this case, the low-pass filter used to isolate the main frequency components of the useful GNSS signal had a cut-off frequency equal to 2 MHz. This design choice preserved the main lobes of the Binary Offset Carrier (BOC) modulation adopted for the Galileo E1c signal.

The results shown in Figure 9 are similar to those discussed above for the GPS L1 C/A modulation. The pure PLL adopted for the processing of the Galileo E1c signal provides improved performance with respect to the Costas loop used for the processing of the GPS L1 C/A modulation. In this respect, the receiver was able to maintain lock for weaker signal conditions. Also, in this case, time domain RIM provided significant performance improvements with respect to standard processing. The selection of the decision threshold, $T_{h}$, allows one to obtain different compromises between loss of efficiency and robustness to jamming. As for the previous case, resilience to jamming improved for large values of $T_{h}$, while the efficiency in the absence of interference progressively decreased. The loss of efficiency was, however, limited and in the order of 1 dB.

### 7.2. Frequency Domain Processing

Frequency domain processing was obtained using the DFT and its fast implementation, the Fast Fourier Transform (FFT), as the first linear transformation, $T_{1}$, and the IDFT for the materialization of $T_{2}$. The size of the two transformations was set here to be equal to 1 ms ($10^{4}$ samples) for the GPS L1 C/A case and to 4 ms ($4\times 10^{4}$) for the processing of the Galileo signals. These values correspond to the length of the code periods of the two GNSS signals.

**Figure 10:**
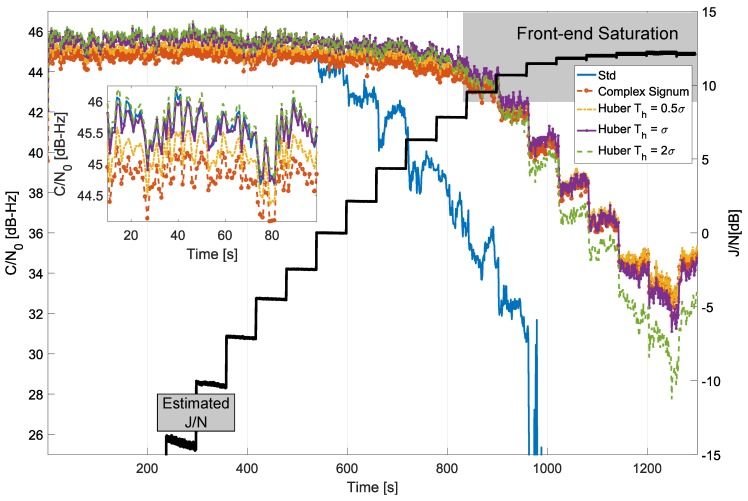
Comparison of $C/N_{0}$ values obtained considering different frequency domain processing schemes. Anechoic chamber test with GPS signals.

The results obtained using the frequency domain RIM are provided in Figure 10 for the GPS case. Similar to the time domain case, RIM provided significant improvements with respect to standard processing. In this case, all frequency domain mitigation techniques allowed the receiver to maintain signal lock for the whole duration of the experiment.

The case with $T_{h}=2\sigma$ had the best efficiency at the expense of a reduced robustness to jamming. On the other side, the complex signum non-linearity provided the highest robustness and the lowest efficiency.

**Figure 11:**
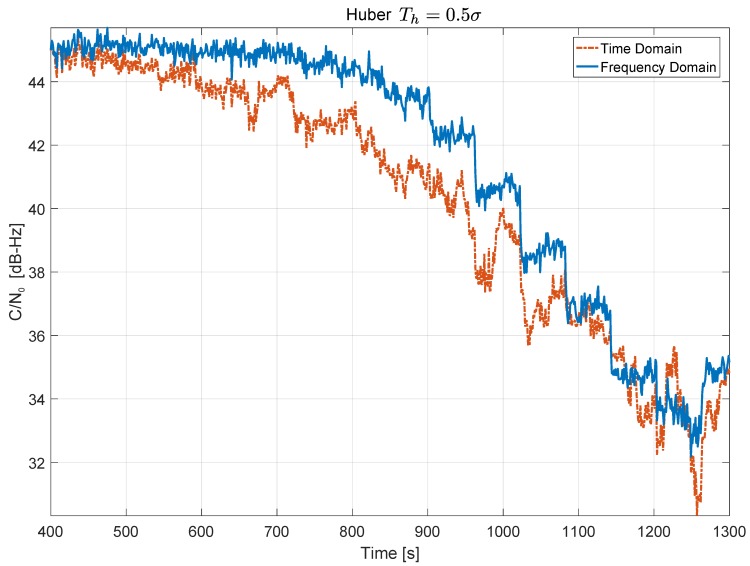
Comparison between $C/N_{0}$ time series obtained using time domain and frequency domain processing. Anechoic chamber test with GPS signals. In both cases, Huber’s non-linearity with $T_{h}=0.5\sigma$ was used.

In this case, frequency domain processing was more effective than its time domain counterpart. This fact can be clearly seen in Figure 11 that compares $C/N_{0}$ time series obtained using time domain and frequency domain processing for $T_{h}=0.5\sigma$.

The $C/N_{0}$ curve obtained using frequency domain processing is practically always above the curve obtained using time domain processing. The benefits of frequency domain processing are more evident for medium levels of jamming, before front-end saturation. In such cases, a gain of about 2 dB can be observed. The improved performance of frequency domain processing is due to the fact that the jamming signal affects less samples when projected into the frequency domain than when filtered in the time domain. Increased performance is however paid by the implementation cost of the FFT operations.

**Figure 12:**
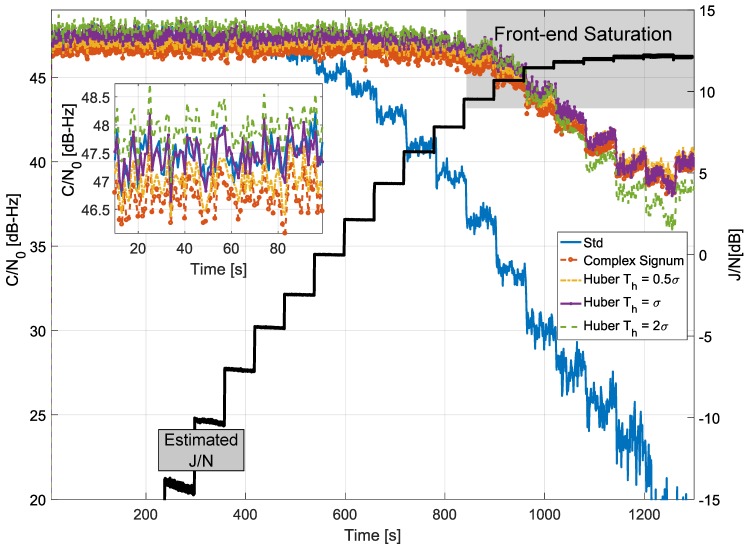
Comparison of $C/N_{0}$ values obtained considering different frequency domain processing schemes. Anechoic chamber test with Galileo E1c signals.

The results obtained processing the Galileo E1c signal are provided in Figure 12. The results are consistent with the previous findings where different compromises between robustness and efficiency are obtained by varying the decision threshold, $T_{h}$.

## 8. Conclusions

Interference mitigation is crucial to protect GNSS from both intentional or unintentional jamming signals. Due to the widespread use of GNSS technology for personal use, but also in managing critical infrastructures, there is a need to develop techniques that make GNSS resilient to those relatively easy to occur, threats. This paper presented RIM, an interference mitigation framework that exploits the sparsity of jamming signals in a convenient domain (e.g., time or frequency) before correlation with the local codes. The methodology leverages results in robust statistics and expands it when appropriate, for instance to derive the Huber’s non-linearity for complex-valued signals. This paper provided analytical expressions for the loss of efficiency, that is, the degradation of performance caused by the proposed method under nominal conditions where the jamming signal is not present, showing negligible losses. The RIM approach has the desirable feature of avoiding the estimation of the jamming signal waveform, which drastically reduces its usability. Performance was analysed using both synthetic data and an experimental setup, validating the remarkable mitigation results with respect to state-of-the-art techniques. 

## Figures and Tables

**Table 1:** Simulation parameters used for the evaluation of the loss, $L_{0}\left(T_{h}\right)$, for the case of frequency domain processing.

Parameter	Value
Sampling Frequency	$f_{s}=4$ MHz
Signal	GPS L1 C/A
Integration Time	1 ms
$C/N_{0}$	40 dB-Hz
DFT size	4000 samples
No. of Simulation Runs	$4\times 10^{5}$

**Table 2:** Parameters of the NI PXI-5663 front-end adopted for the data collection.

Parameter	Value
Centre Frequency	1575.42 MHz
Sampling Frequency	10 MHz
Sampling Type	Complex I&Q
Number of bits	16

**Table 3:** Summary of the parameters adopted for the processing of the GPS L1 C/A and Galileo E1c signals.

Parameter	GPS L1 C/A	Galileo E1c
Coherent integration time	1 ms	4 ms
Coherent integration time after bit/secondary code synchronization	20 ms	20 ms
DLL order	2	2
DLL bandwidth	5 Hz	5 Hz
PLL order	3	3
PLL bandwidth	10 Hz	10 Hz

## Appendix A. Moments of the Samples Processed with Huber’s Non-Linearity

In this appendix, the first two moments of complex samples processed with Huber’s non-linearity are derived under the assumptions of Gaussian noise and for weak signal conditions. The two moments derived here are used to compute loss (24).

Under the hypothesis of input Gaussian noise, the pdf of y[n] can be written as

(A1) $$f_{y}\left(y\left[n\right]\right)=\frac{1}{2\pi \sigma^{2}}\exp\left\{-\frac{1}{2\sigma^{2}}{\left|y\left[n\right]-Ae^{j\theta_{0}\left[n\right]}\right|}^{2}\right\},$$

where $A=\sqrt{C}$ models the signal amplitude, $\theta_{0}$ the signal phase and $\sigma^{2}$ the variance in the real and imaginary parts of the noise affecting the input samples. In particular,

$$E\left\{y[n]\right\}=Ae^{j\theta_{0}\left[n\right]}.$$

In the following text, the dependency on *n*, the time index, is omitted for ease of notation. Equation (A1) can be written in polar form as

(A2) $$f_{y}\left(r,\theta \right)=\frac{r}{2\pi \sigma^{2}}\exp\left\{-\frac{A^{2}+r^{2}}{2\sigma^{2}}\right\}\exp\left\{\frac{Ar}{\sigma^{2}}\cos\left(\theta -\theta_{0}\right)\right\},$$

where r=|y[n]| and θ=∠y[n]. Also, in this case, the dependency on *n* has been omitted for ease of notation. We are interested in determining $E\left\{\psi_{H}\left(y\left[n\right]\right)\right\}$ and $E\left\{\psi_{H}^{2}\left(y\left[n\right]\right)\right\}$.

The first moment of $\psi_{H}\left(y\left[n\right]\right)$ is given by the sum of two terms, corresponding to the two branches in (11):

(A3) $$\begin{array}{cc} E\left\{\psi_{H}\left(y\left[n\right]\right)\right\} & =\int_{0}^{T_{h}}\int_{-\pi }^{\pi }re^{j\theta }\frac{r}{2\pi \sigma^{2}}\exp\left\{-\frac{A^{2}+r^{2}}{2\sigma^{2}}\right\}\exp\left\{\frac{Ar}{\sigma^{2}}\cos\left(\theta -\theta_{0}\right)\right\}d\theta dr\\ & +T_{h}\int_{T_{h}}^{+\infty }\int_{-\pi }^{\pi }e^{j\theta }\frac{r}{2\pi \sigma^{2}}\exp\left\{-\frac{A^{2}+r^{2}}{2\sigma^{2}}\right\}\exp\left\{\frac{Ar}{\sigma^{2}}\cos\left(\theta -\theta_{0}\right)\right\}d\theta dr\\ & =I_{\psi ,1}\left(T_{h}\right)+I_{\psi ,2}\left(T_{h}\right). \end{array}$$

The first term in (A3) can be computed by isolating the mean phase term, $\theta_{0}$ and computing, at first, the integral with respect to the phase variable. In particular,

(A4) $$I_{\psi ,1}\left(T_{h}\right)=e^{j\theta_{0}}\int_{0}^{T_{h}}\frac{r^{2}}{2\pi \sigma^{2}}\exp\left\{-\frac{A^{2}+r^{2}}{2\sigma^{2}}\right\}\int_{-\pi }^{\pi }e^{j(\theta -\theta_{0})}\exp\left\{\frac{Ar}{\sigma^{2}}\cos\left(\theta -\theta_{0}\right)\right\}d\theta dr.$$

The integral with respect to θ can be simplified by noting that the imaginary component of such integral is zero. This is due to the fact that the imaginary part of the function under integral is odd and thus leads to a zero integral. Using the change in the variable $\varphi =\theta -\theta_{0}$ and noting that the function under the integral is a periodic of 2π, it is finally possible to express the integral with respect to the phase variable as $I_{1}\left(\frac{Ar}{\sigma^{2}}\right)$, where $I_{1}\left(\cdot \right)$ is the modified Bessel function of first kind and order 1 ([33] p. 376). This result directly follows on from the integral definition of the modified Bessel function of the first kind (see Equation (9.6.19) of [33]).

In this way,

(A5) $$I_{\psi ,1}\left(T_{h}\right)=e^{j\theta_{0}}\int_{0}^{T_{h}}\frac{r^{2}}{\sigma^{2}}\exp\left\{-\frac{A^{2}+r^{2}}{2\sigma^{2}}\right\}I_{1}\left(\frac{Ar}{\sigma^{2}}\right)dr.$$

The (A5) integral can be computed in closed form using the definition of generalized Marcum Q-function [40]. In particular,

(A6) $$I_{\psi ,1}\left(T_{h}\right)=Ae^{j\theta_{0}}\left[1-Q_{2}\left(\frac{A}{\sigma },\frac{T_{h}}{\sigma }\right)\right],$$

where $Q_{2}\left(\cdot ,\cdot \right)$ is the generalized Marcum Q-function of the second order [40].

Under weak signal conditions, i.e., for $\frac{A}{\sigma }\ll 1$, the generalized Marcum Q-function of the second order can be approximated as

(A7) $$Q_{2}\left(\frac{A}{\sigma },\frac{T_{h}}{\sigma }\right)\approx e^{-\frac{T_{h}^{2}}{2\sigma^{2}}}\left(1+\frac{T_{h}^{2}}{2\sigma^{2}}\right)\;\mathrm{for}\;\frac{A}{\sigma }\to 0$$

and

(A8) $$I_{\psi ,1}\left(T_{h}\right)\approx Ae^{j\theta_{0}}\left[1-e^{-\frac{T_{h}^{2}}{2\sigma^{2}}}\left(1+\frac{T_{h}^{2}}{2\sigma^{2}}\right)\right].$$

The second term in (A3) can be computed by starting from the integral with respect to *r*. In this respect, $I_{\psi ,2}\left(T_{h}\right)$ can be expressed as

(A9) $$I_{\psi ,2}\left(T_{h}\right)=T_{h}e^{j\theta_{0}}\int_{-\pi }^{\pi }e^{j\varphi }\exp\left\{-\frac{A^{2}\sin^{2}\varphi }{2\sigma^{2}}\right\}\int_{T_{h}}^{+\infty }\frac{r}{2\pi \sigma^{2}}\exp\left\{-\frac{{\left(r-A\cos\varphi \right)}^{2}}{2\sigma^{2}}\right\}drd\varphi ,$$

where the change in the variable $\varphi =\theta -\theta_{0}$ is performed. The integration limits are changed, taking into account that all the functions of φ appearing in $I_{\psi ,2}\left(T_{h}\right)$ are periodics of 2π.

The integral with respect to *r* can be computed using the properties of Gaussian random variables (the function inside the second integral in (A9) can be re-conducted to the pdf of a real Gaussian variable with mean Acos(φ) and variance $\sigma^{2}$). It is possible to show that

(A10) $$\begin{array}{c} \int_{T_{h}}^{+\infty }\frac{r}{2\pi \sigma^{2}}\exp\left\{-\frac{{\left(r-A\cos\varphi \right)}^{2}}{2\sigma^{2}}\right\}dr\\ =\frac{1}{2\pi }\exp\left\{-\frac{{\left(T_{h}-A\cos\varphi \right)}^{2}}{2\sigma^{2}}\right\}+\frac{1}{2\sqrt{2\pi }}\frac{A}{\sigma }\cos\varphi \cdot \mathrm{erfc}\left(\frac{T_{h}-A\cos\varphi }{\sqrt{2}\sigma }\right) \end{array}.$$

where erfc(·) is the complementary error function ([33] p. 296):

(A11) $$\mathrm{erfc}\left(z\right)=\frac{2}{\sqrt{\pi }}\int_{z}^{+\infty }e^{-t^{2}}dt.$$

Using (A10), (A9) can be expressed as

(A12) $$\begin{array}{cc} I_{\psi ,2}\left(T_{h}\right) & =T_{h}e^{j\theta_{0}}\int_{-\pi }^{\pi }e^{j\varphi }\exp\left\{-\frac{A^{2}\sin^{2}\varphi }{2\sigma^{2}}\right\}\frac{1}{2\pi }\exp\left\{-\frac{{\left(T_{h}-A\cos\varphi \right)}^{2}}{2\sigma^{2}}\right\}d\varphi \\ & +T_{h}e^{j\theta_{0}}\int_{-\pi }^{\pi }e^{j\varphi }\exp\left\{-\frac{A^{2}\sin^{2}\varphi }{2\sigma^{2}}\right\}\frac{1}{2\sqrt{2\pi }}\frac{A}{\sigma }\cos\varphi \cdot \mathrm{erfc}\left(\frac{T_{h}-A\cos\varphi }{\sqrt{2}\sigma }\right)d\varphi \\ & =I_{\psi ,2a}\left(T_{h}\right)+I_{\psi ,2b}\left(T_{h}\right) \end{array}.$$

The first integral in (A12) can be expressed in closed form using the definition of the modified Bessel functions of the first kind:

(A13) $$I_{\psi ,2a}\left(T_{h}\right)=T_{h}e^{j\theta_{0}}\exp\left\{-\frac{T_{h}^{2}+A^{2}}{2\sigma^{2}}\right\}I_{1}\left(\frac{AT_{h}}{\sigma^{2}}\right).$$

The second integral, $I_{\psi ,2b}\left(T_{h}\right)$, does not seem to admit a simple closed form expression. However, the weak signal assumption can be exploited, and $\mathrm{erfc}\left(\frac{T_{h}-A\cos\varphi }{\sqrt{2}\sigma }\right)$ can be expanded in Taylor series, leading to the following approximation:

(A14) $$\begin{array}{cc} \mathrm{erfc}\left(\frac{T_{h}-A\cos\varphi }{\sqrt{2}\sigma }\right) & \approx \mathrm{erfc}\left(\frac{T_{h}}{\sqrt{2}\sigma }\right)-{\left.\frac{d}{dx}\mathrm{erfc}\left(x\right)\right|}_{x=\frac{T_{h}}{\sqrt{2}\sigma }}\cdot \frac{A}{\sqrt{2}\sigma }\cos\varphi .\\ & =\mathrm{erfc}\left(\frac{T_{h}}{\sqrt{2}\sigma }\right)+\sqrt{\frac{2}{\pi }}e^{-\frac{T_{h}^{2}}{2\sigma^{2}}}\frac{A}{\sigma }\cos\varphi \end{array}.$$

In this way,

(A15) $$\begin{array}{cc} I_{\psi ,2b}\left(T_{h}\right) & =T_{h}e^{j\theta_{0}}\mathrm{erfc}\left(\frac{T_{h}}{\sqrt{2}\sigma }\right)\int_{-\pi }^{\pi }e^{j\varphi }\exp\left\{-\frac{A^{2}\sin^{2}\varphi }{2\sigma^{2}}\right\}\frac{1}{2\sqrt{2\pi }}\frac{A}{\sigma }\cos\varphi d\varphi \\ & +T_{h}e^{j\theta_{0}}e^{-\frac{T_{h}^{2}}{2\sigma^{2}}}\int_{-\pi }^{\pi }e^{j\varphi }\exp\left\{-\frac{A^{2}\sin^{2}\varphi }{2\sigma^{2}}\right\}\frac{1}{2\pi }\frac{A^{2}}{\sigma^{2}}\cos^{2}\varphi . \end{array}$$

Since the second term in (A15) is proportional to $A^{2}/\sigma^{2}$, it can be neglected using the weak signal assumption. Moreover,

$$\exp\left\{-\frac{A^{2}\sin^{2}\varphi }{2\sigma^{2}}\right\}\approx 1\;\mathrm{for}\frac{A}{\sigma^{2}}\to 0.$$

In this way,

(A16) $$\begin{array}{cc} I_{\psi ,2b}\left(T_{h}\right) & \approx T_{h}e^{j\theta_{0}}\mathrm{erfc}\left(\frac{T_{h}}{\sqrt{2}\sigma }\right)\frac{1}{2\sqrt{2\pi }}\frac{A}{\sigma }\int_{-\pi }^{\pi }e^{j\varphi }\cos\varphi d\varphi \\ & =\frac{T_{h}A}{\sigma }e^{j\theta_{0}}\frac{\sqrt{\pi }}{2\sqrt{2}}\mathrm{erfc}\left(\frac{T_{h}}{\sqrt{2}\sigma }\right). \end{array}$$

Using (A16) and (A13), it is finally possible to compute the second term in (A3):

(A17) $$I_{\psi ,2}\left(T_{h}\right)=T_{h}e^{j\theta_{0}}\left[\exp\left\{-\frac{T_{h}^{2}+A^{2}}{2\sigma^{2}}\right\}I_{1}\left(\frac{AT_{h}}{\sigma^{2}}\right)+\frac{A}{\sigma }\frac{1}{2}\sqrt{\frac{\pi }{2}}\mathrm{erfc}\left(\frac{T_{h}}{\sqrt{2}\sigma }\right)\right].$$

Equation (A17) can be further simplified by assuming that $\frac{AT_{h}}{\sigma^{2}}$ is small. Using Equation (9.6.10) in ([33], p. 375), it is possible to approximate $I_{1}\left(\frac{AT_{h}}{\sigma^{2}}\right)$ as $\frac{1}{2}\frac{AT_{h}}{\sigma^{2}}$ and

(A18) $$I_{\psi ,2}\left(T_{h}\right)\approx Ae^{j\theta_{0}}\left[\frac{T_{h}^{2}}{2\sigma^{2}}\exp\left\{-\frac{T_{h}^{2}}{2\sigma^{2}}\right\}+\frac{\sqrt{\pi }}{2}\frac{T_{h}}{\sqrt{2}\sigma }\mathrm{erfc}\left(\frac{T_{h}}{\sqrt{2}\sigma }\right)\right].$$

Finally, combining (A18) and (A8):

(A19) $$\begin{array}{cc} E\left\{\psi_{H}\left(y\left[n\right]\right)\right\} & \approx Ae^{j\theta_{0}}\left[1-e^{-\frac{T_{h}^{2}}{2\sigma^{2}}}\left(1+\frac{T_{h}^{2}}{2\sigma^{2}}\right)+\frac{T_{h}^{2}}{2\sigma^{2}}e^{-\frac{T_{h}^{2}}{2\sigma^{2}}}+\frac{\sqrt{\pi }}{2}\frac{T_{h}}{\sqrt{2}\sigma }\mathrm{erfc}\left(\frac{T_{h}}{\sqrt{2}\sigma }\right)\right]\\ & =E\left\{y[n]\right\}\left[1-e^{-\frac{T_{h}^{2}}{2\sigma^{2}}}+\frac{\sqrt{\pi }}{2}\frac{T_{h}}{\sqrt{2}\sigma }\mathrm{erfc}\left(\frac{T_{h}}{\sqrt{2}\sigma }\right)\right] \end{array}$$

where the approximation is valid under weak signal conditions. From (A19), it emerges that $E\left\{\psi_{H}\left(y\left[n\right]\right)\right\}$ is proportional to the mean of y[n] and that the amplitude scaling factor only depends on $T_{h}/\sigma$, the normalized decision threshold.

As for the previous case, the second moment of $\psi_{H}\left(y\left[n\right]\right)$ is given by two terms:

(A20) $$\begin{array}{cc} E\left\{|\psi_{H}{\left(y\left[n\right]\right)|}^{2}\right\} & =\int_{0}^{T_{h}}\int_{-\pi }^{\pi }r^{2}\frac{r}{2\pi \sigma^{2}}\exp\left\{-\frac{A^{2}+r^{2}}{2\sigma^{2}}\right\}\exp\left\{\frac{Ar}{\sigma^{2}}\cos\left(\theta -\theta_{0}\right)\right\}d\theta dr\\ & +T_{h}^{2}\int_{T_{h}}^{+\infty }\int_{-\pi }^{\pi }\frac{r}{2\pi \sigma^{2}}\exp\left\{-\frac{A^{2}+r^{2}}{2\sigma^{2}}\right\}\exp\left\{\frac{Ar}{\sigma^{2}}\cos\left(\theta -\theta_{0}\right)\right\}d\theta dr\\ & =\int_{0}^{T_{h}}\frac{r^{3}}{\sigma^{2}}\exp\left\{-\frac{A^{2}+r^{2}}{2\sigma^{2}}\right\}\frac{1}{2\pi }\int_{-\pi }^{\pi }\exp\left\{\frac{Ar}{\sigma^{2}}\cos\varphi \right\}d\varphi dr\\ & +T_{h}^{2}\int_{T_{h}}^{+\infty }\frac{r}{\sigma^{2}}\exp\left\{-\frac{A^{2}+r^{2}}{2\sigma^{2}}\right\}\frac{1}{2\pi }\int_{-\pi }^{\pi }\exp\left\{\frac{Ar}{\sigma^{2}}\cos\varphi \right\}d\varphi dr\\ & =\int_{0}^{T_{h}}\frac{r^{3}}{\sigma^{2}}\exp\left\{-\frac{A^{2}+r^{2}}{2\sigma^{2}}\right\}I_{0}\left(\frac{Ar}{\sigma^{2}}\right)dr\\ & +T_{h}^{2}\int_{T_{h}}^{+\infty }\frac{r}{\sigma^{2}}\exp\left\{-\frac{A^{2}+r^{2}}{2\sigma^{2}}\right\}I_{0}\left(\frac{Ar}{\sigma^{2}}\right)dr. \end{array}$$

Under weak signal conditions, the first integral in the last equality of (A20) can be approximated as

(A21) $$\begin{array}{c} \int_{0}^{T_{h}}\frac{r^{3}}{\sigma^{2}}\exp\left\{-\frac{A^{2}+r^{2}}{2\sigma^{2}}\right\}I_{0}\left(\frac{Ar}{\sigma^{2}}\right)dr\approx \int_{0}^{T_{h}}\frac{r^{3}}{\sigma^{2}}\exp\left\{-\frac{r^{2}}{2\sigma^{2}}\right\}dr\\ =-{\left.r^{2}e^{-\frac{r^{2}}{2\sigma^{2}}}\right|}_{0}^{T_{h}}+\int_{0}^{T_{h}}2re^{-\frac{r^{2}}{2\sigma^{2}}}dr=-T_{h}^{2}e^{-\frac{T_{h}^{2}}{2\sigma^{2}}}+2\sigma^{2}{\left.e^{-\frac{r^{2}}{2\sigma^{2}}}\right|}_{0}^{T_{h}}\\ =2\sigma^{2}-\left(T_{h}^{2}+2\sigma^{2}\right)e^{-\frac{T_{h}^{2}}{2\sigma^{2}}}. \end{array}$$

The second integral in (A20) corresponds to the definition of the Marcum Q-function and is equal to

(A22) $$T_{h}^{2}\int_{T_{h}}^{+\infty }\frac{r}{\sigma^{2}}\exp\left\{-\frac{A^{2}+r^{2}}{2\sigma^{2}}\right\}I_{0}\left(\frac{Ar}{\sigma^{2}}\right)dr=T_{h}^{2}Q_{1}\left(\frac{A}{\sigma };\frac{T_{h}}{\sigma }\right)\approx T_{h}^{2}e^{-\frac{T_{h}^{2}}{2\sigma^{2}}},$$

where the last approximation is valid for weak signal conditions.

Using (A21) and (A22), it follows that

(A23) $$E\left\{|\psi_{H}{\left(y\left[n\right]\right)|}^{2}\right\}=2\sigma^{2}\left[1-e^{-\frac{T_{h}^{2}}{2\sigma^{2}}}\right]\;\mathrm{for}\frac{A}{\sigma^{2}}\to 0.$$

It is noted that second moment (A23) is proportional to $\sigma^{2}$, whereas the first moment (A19) is proportional to *A*. Since we are considering weak signal conditions, $\sigma^{2}\gg A^{2}$ and

(A24) $$E\left\{|\psi_{H}{\left(y\left[n\right]\right)|}^{2}\right\}\gg {\left|E\left\{\psi (y[n])\right\}\right|}^{2}.$$

For this reason, under weak signal conditions, the second moment can be used to approximate the variance of the samples pre-processed with Huber’s non-linearity:

(A25) $$Var\left\{\psi_{H}\left(y\left[n\right]\right)\right\}=E\left\{|\psi_{H}{\left(y\left[n\right]\right)|}^{2}\right\}-{\left|E\left\{\psi_{H}\left(y\left[n\right]\right)\right\}\right|}^{2}\approx E\left\{|\psi_{H}{\left(y\left[n\right]\right)|}^{2}\right\}=2\sigma^{2}\left[1-e^{-\frac{T_{h}^{2}}{2\sigma^{2}}}\right].$$
